# Reliable improvements in participation in low-impact sports following implantation of a patellofemoral inlay arthroplasty at mid-term follow-up

**DOI:** 10.1007/s00167-020-06245-5

**Published:** 2020-08-26

**Authors:** Jonas Pogorzelski, Marco-Christopher Rupp, Conrad Ketzer, Matthias Cotic, Patricia Lutz, Saskia Beeck, Andreas B. Imhoff, Matthias J. Feucht

**Affiliations:** 1 Department of Orthopedic Sports Medicine, Technical University of Munich, Klinikum rechts der Isar, Ismaninger Str. 22, 81675 Munich, Germany; 2 Department of Orthopaedics and Trauma Surgery, Medical Center, Faculty of Medicine, Albert-Ludwigs-University of Freiburg, Freiburg, Germany

**Keywords:** Patellofemoral, Knee, Patellofemoral osteoarthritis, Patellofemoral arthroplasty, Patellofemoral resurfacing, Trochlear, Return to sports, Return to activity

## Abstract

**Purpose:**

The aim of this study was, to investigate the rate of return to sports (RTS) and physical activity after implantation of PFIA and to identify factors predictive of improved postoperative sporting ability.

**Methods:**

Sixty-two patients with a mean age of 46 ± 11 years, who underwent implantation of PFIA at the senior authors’ institution, were enrolled. They were prospectively evaluated preoperatively and at a minimum of 2 years postoperatively with a mean follow-up of 60 ± 25 months. Clinical outcomes, return to sports and activity, type of sport or activity, subjective satisfaction, and frequency were evaluated by questionnaire.

**Results:**

The transformed overall Western Ontario and McMaster Universities Osteoarthritis Index (WOMAC) score improved from 67 ± 16 to 77 ± 19 (*p = *0.003), Tegner activity scale results improved from 3 ± 2 points to 4 ± 1 points (*p* < 0.001), and scores on the visual analog scale (VAS) pain scale decreased from 6 ± 2 points to 3 ± 2 points (*p* < 0.001). The sports frequency increased from 1 ± 2 sessions to 2 ± 1 sessions per week (*p* = 0.001). Ninety-four percent of the patients who did not fail could return to the same or higher level of sports, with 74% of the patients reporting an improved ability to perform sports. No preoperative factors could be detected to significantly influence RTS after surgery.

**Conclusions:**

PFIA is a valid treatment option for the active patient with end-stage isolated patellofemoral OA. Reliable improvements in knee function, pain, and participation in low-impact sports were found.

**Level of evidence:**

IV.

## Introduction

Patellofemoral inlay arthroplasty (PFIA) is considered as a viable treatment option in the case of end-stage isolated patellofemoral osteoarthritis [7, 10, 11, 18]. Alongside innovation and improvement in PFA models, recent advances in surgical technique, technology, and implant design of patellofemoral inlay arthroplasties (PFIA) have improved the clinical outcomes and survival [10, 27]. Consequently, recent reports in the literature propose PFIA as a treatment for the young and active patient collective [6, 31]. This particular patient cohort, however, has high expectations concerning the postoperative level of physical activity and return to sports.

Although a rising number of studies evaluated the postoperative outcome following PFIA, there is still a lack of information about the postoperative return to sport and physical activity [12, 22]. While multiple authors reported promising results concerning the return to activities following unicompartmental knee arthroplasty (UKA) of the tibiofemoral joint [14, 20, 30], only limited data exist on the return to activity following patellofemoral arthroplasty [24].

Therefore, the purpose of this longitudinal, retrospective, and minimum 2-year follow-up study was to specifically investigate the rate of return to sports and physical activity after implantation of PFIA for patients suffering from isolated patellofemoral osteoarthritis. The secondary purpose was to investigate risk factors associated with an inability to return to sports. We hypothesized that the implantation of PFIA would result in a high return to sports rate with an improved postoperative level of activity, and we would identify risk factors which prevented return to the same level of sports and activity.

## Methods

### Study population

This was an Institutional-Review-Board approved level IV retrospective outcome study of prospectively collected data. Review of our institutional data bank was performed to identify patients meeting the following inclusion criteria: patients who underwent PFIA with or without concomitant procedures to address patellofemoral instability and/or malalignment at the senior author’s institution, with a minimum of 2-year postoperative follow-up. Informed consent was obtained by each patient. Patients were excluded, if they deceased during follow-up, if they had additional knee surgery, unrelated to the patellofemoral joint on the ipsilateral knee, and if they were converted to a total knee arthroplasty, to precisely evaluate return to sports in a successful PFIA treatment.

**Fig. 1:**
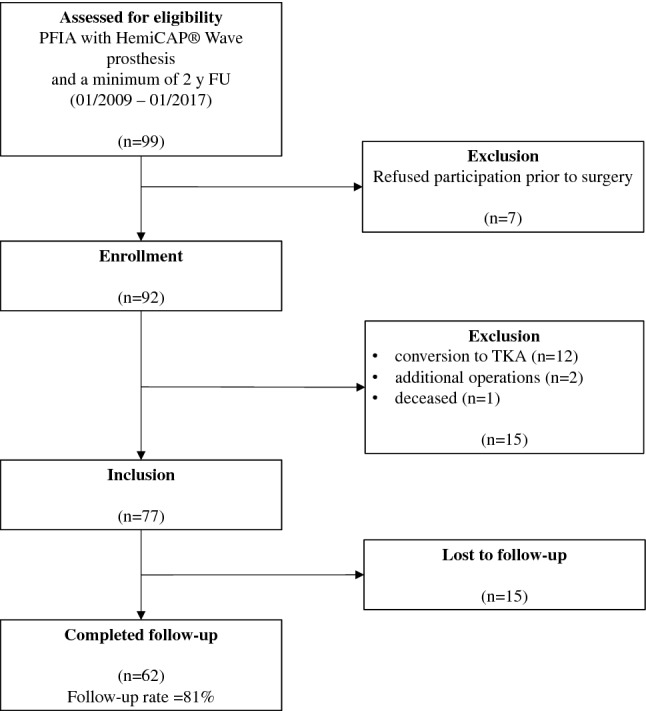
Flowchart of the patient selection and evaluation process

Between January 2009 and January 2017, review of our institutional database identified 99 patients who underwent implantation of PFIA, with a minimum 2-year postoperative time interval. Seven patients refused to participate prior to their surgery and, therefore, were excluded. This left a study population of 92 patients. Of those, another 15 patients were excluded for conversion to TKA, additional operations on the ipsilateral knee and death during follow-up (Fig. 1). The remaining 77 patients could be included. Despite our best efforts to attain follow-up, 15 patients could not be reached for follow-up evaluation and thus considered lost to follow up. Therefore, final data analysis was available for 62 patients (26 men, 36 women; 81% follow-up) (Fig. 1).

Mean age at the time of index surgery was 46 ± 11 years with a mean postoperative follow-up of 60 ± 25 months. Eighteen patients (29%) underwent concomitant procedures addressing patellofemoral malalignment and 20 patients (32%) received patellar resurfacing. Detailed characteristics of the patient collective and information on prevalence of investigated preoperative factors in the study population can be found in Table 1.

**Table 1:** Overall data and descriptive analysis of the entire patient group

Preoperative and perioperative patient characteristics	*N* = 62 (100%)
Sex distribution	
Male	26 (42%)
Female	36 (58%)
Age (years)	46 ± 11
Body mass index (kg/m^2^)	27 ± 5
Follow-up (months)	73 ± 25
Concomitant procedures	
Yes	18 (29%)
Osteotomy of the tibial tubercle (*n* = 2)	
Isolated MPFL reconstruction (*n* = 5)	
Isolated DFO (*n* = 5)	
Combined osteotomy of the tibial tubercle and MPFL reconstruction (*n* = 4)	
Combined DFO and MPFL reconstruction (*n* = 1)	
Combined DFO, MPFL reconstruction, tibial tubercle (*n* = 1)	
No	44 (71%)
Patellar resurfacing	
Yes	20 (32%)
No	42 (68%)

Age, body mass index, and follow-up are given as means ± standard deviation

*N* number of patients

%, percent. kg/m^2^, kilograms per square meter

### Indication

PFIA was indicated in patients with isolated disabling patellofemoral osteoarthritis OA (grade III–IV Kellgren–Lawrence) or chondral defects (grade III–IV Outerbridge) refractory to conservative treatment and/or failed prior surgical treatment. Based on an established treatment algorithm which has been published recently, isolated PFIA was performed in patients without patellofemoral instability or patellofemoral malalignment. In case of symptomatic patellofemoral instability or malalignment (tibial tuberosity trochlear groove distance > 20 mm or < 8 mm, Caton-Deschamps Index > 1.2 or < 0.8, lateral patellar tilt > 5°, mechanical valgus or varus > 5°, femoral anteversion > 20°, tibial torsion > 40° [7, 10]), concomitant procedures such as reconstruction of the medial patellofemoral ligament (MPFL), distal femoral osteotomy (DFO), and tibial tuberosity transfer were performed. Contraindications for PFIA were symptomatic tibiofemoral OA with pain during activities of daily living, inflammatory arthropathy, chondrocalcinosis, chronic regional pain syndrome, active infection, and fixed loss of knee range of motion.

### Implant design and surgical technique

In all patients, the HemiCAP^®^ Wave Patellofemoral Resurfacing Prosthesis (Arthrosurface, Franklin, MA, USA) was used. The implant consists of a cobalt chrome trochlear component, with titanium surface coating, linked to a titanium taper post, and an optional all-polyethylene patella component. Different implant sizes with varying offsets were available, allowing for a patient-specific treatment.

To protect the medial soft-tissue structures, a lateral parapatellar approach was used as a standard approach. An offset drill guide was used to determine a working axis perpendicular to the central trochlear articular surface with the knee in full extension, confirming defect coverage of the trochlea. A guide pin was advanced into the bone, once the superior and inferior drill guide feet were aligned to the trochlear orientation. The medial/lateral and superior/inferior offsets were calculated using specific instrumentation to ensure appropriate implant geometry. Employing a guide block, the implant bed was reamed three-dimensionally. The screw fixation stud was then inserted into the bone, and the trochlear component was aligned with the adequate offsets onto the implant holder and connected to the taper post of the fixation stud. Using an impactor, the trochlear component was then placed [7].

Consecutively, circumpatellar denervation and debridement of patellar osteophytes was performed in all patients. The patella was resurfaced in patients with patellofemoral incongruence caused by focal osteonecrosis or osteolysis with subchondral bone defects and severe patellar dysplasia. To do so, a drill guide was inserted employing an alignment guide. The superior/inferior and medial/lateral offsets were determined and an implant bed was prepared. The patellar component was then aligned on the implant holder and cemented into the implant bed [7].

### Postoperative rehabilitation

As a part of a structured rehabilitation program, patients were limited to partial weight bearing of 20 kg for 2 weeks. Rehabilitation also included decongestant therapy and mobilization was ensured employing continuous passive motion for the first 2 weeks. Full range of motion was allowed immediately after surgery. Subsequently, weight bearing was increased gradually until full weight bearing was achieved approximately 6 weeks after surgery. Patients were discharged from hospital, when a range of motion of flexion/extension 90/0/0 of the knee joint was reached and they could climb stairs on crutches [7]. Return to low-impact sports was permitted after 3 months, return to high-impact sports after 6 months.

### Clinical evaluation

Clinical outcomes were evaluated comparing the preoperative Western Ontario and McMaster Universities Osteoarthritis Index (WOMAC) [1], the preoperative visual analog scale for pain (VAS), as well as the preoperative Tegner Activity Scale [25] to a minimum of 2-year postoperative follow-up. The WOMAC score was assessed according to the KOOS User`s Guide. Standardized answer options were given as five Likert boxes, and each question got a score from 0 to 4. Transformation included calculating a normalized percentage score (100 indicating no problems and 0 indicating extreme problems) for each subscale. Return to sports and activity, type of sport, and frequency of the activity (defined as sessions per week) were evaluated by questionnaire. Changes in the level of sports were evaluated using an ordinal scale, which differentiates the level of sports between “daily activity”, “recreational sports”, and “professional sports” preoperatively and postoperatively. To provide a concise understanding of the sportive activity in the patient collective, the various types of sports were asked preoperatively and the return to each sport or activity was evaluated postoperatively. The subjective postoperative ability to perform sports was qualitatively rated on an ordinal scale consisting of “improved”, “equal to preoperative state”, or “deteriorated” sporting activity. To differentiate the reasons for subjectively “deteriorated” sporting activity postoperatively, an additional question investigating the reason for deterioration was asked, with the following response options being provided: 1 due to the operated knee; 2 due to other physical problems not related to the operated knee; 3 due to non-physical personal reasons such as shortage of time due to obligations in family, professional career, etc.

The association between preoperative characteristics and improved subjective sporting ability postoperatively was assessed performing a subgroup analysis. The size of our study population statistically limited the number of risk factors to be evaluated, since repeatedly testing an excessive number of factors on a single dataset predisposes for the occurrence of Type 1 (false-positive) errors. Therefore, we selected only the following preoperative factors a priori for assessment of our secondary hypothesis in this study: constitutional factors (BMI, age, and sex), the influence of above -mentioned concomitant procedures addressing patellofemoral instability or malalignment, and the influence of patellar resurfacing. Success was defined as patients choosing “improved” on the ordinal scale evaluating their satisfaction with the postoperative sporting activity.

### Statistical analysis

Data analysis was performed using SPSS software version 22.0 (IBM-SPSS, New York, USA). A minimal clinically important difference on the total WOMAC score of 15 points was determined in a previous validation study for patients following knee arthroplasty. An a priori power analysis was calculated with a difference to detect of 15 points and a standard deviation of 10 points in the WOMAC score. A sample size of 20 patients with *α* = 0.05 and *β* = 0.2 for a power of 80% was established. Normally distributed data are reported as mean ± standard deviation, whereas non-normally distributed data are reported as median and range (interquartile range, IQR, from the 25th to the 75th percentile). In non-normally distributed data, the non-parametric Wilcoxon test for two related samples was used to compare preoperative and postoperative values of each outcome parameter. The non-parametric Mann–Whitney *U* test for two independent samples was used to compare failures and survivors. The level of significance was set at *p* < 0.05.

## Results

### Clinical outcome

The preoperative scores were compared to scoring at a final follow-up of 60 ± 25 month. The overall transformed WOMAC scoring improved from 67 ± 16 to 77 ± 19 (*p = *0.003), the Tegner activity scale improved from 3 ± 2 points to 4 ± 1 points (*p* < 0.001), and the pain intensity assessed with the VAS pain scale declined from 6 ± 2 points to 3 ± 2 points (*p* < 0.001).

### Results of return to sports/activity query

A comprehensive overview over the sport disciplines across the patient collective can be found in Table 3. While preoperatively, a large percentage of the patients participated in low-impact sports such as biking (53%), hiking (16%), or swimming (15%), only few patients reported to participate in contact or pivot sports such as tennis (2%) or baseball (2%). Prior to surgery, 11 patients (18%) only participated in daily activities, while 50 patients (81%) participated in recreational sports and one patient (2%) competed on a professional level. A total of 46 (74%) patients reported a subjectively improved ability to participate in sports and activities, while 10 (16%) patients reported an equal ability to participate in their sports and 6 (10%) patients reported a reduced ability to participate in their sports. In the patient subgroup that reported a reduced subjective satisfaction in their sport, five (83%) patients attributed the deterioration to the operated knee, while one (17%) patient accredited it to non-physical personal reasons. The level of sports participation comprising “daily activities”, “recreational sports”, and “professional sports” improved across the patient collective, with 15 (24%) patients exercising their sport at a higher level, 43 (70%) patients practicing their sport at the same level, and 4 (6%) patients having downscaled to a lower level of sports. This results in a RTS rate—defined as return to equal or higher level of sports postoperatively compared to preoperatively—of 94% in our series. Considering the 14 patients who failed as additionally “failed to return to sport”, the RTS rate drops to 76%. In general, frequency of sports and activities increased from 1 ± 2 sessions per week preoperatively to 2 ± 1 sessions at final follow-up (*p* = 0.001).

### Factors predictive for an improvement of postoperative subjective sporting ability

The subgroup analysis investigating possible factors predictive for improvement of postoperative sporting ability showed that constitutional factors such as sex, BMI, and age did not significantly influence the postoperative subjective ability to perform sports. Furthermore, neither concomitant procedures nor patellar resurfacing influenced the postoperative ability to perform sports in a statistically significantly way. Results of the analysis are shown in Table 2.

**Table 2:** Comparison of preoperative and perioperative characteristics between successful and failed improvement of subjective sporting ability

	Improvement (*n* = 46)	Failed improvement (*n* = 16)	Significance
Gender distribution			
Male	22 (48%)	4 (25%)	*p* = 0.111^#^
Female	24 (52%)	12 (75%)	
Procedures			*p* = 0.386^#^
Isolated	34 (74%)	10 (63%)	
Combined	12 (26%)	6 (37%)	
Patellar resurfacing			*p* = 0.180^#^
No	29 (63%)	13 (81%)	
Yes	17 (37%)	3 (19%)	
Age (years)	46 (38–55)	46 (41–53)	*p* = 0.841^#^
Body mass index (kg/m^2^)	28 (24–29)	28 (25–31)	*p* = 0.682^#^

Improvement was defined as patients reporting “improved”, failed improvement as “equal”, or “deteriorated” subjective sporting ability

Values are given as median and interquartile range (25th–75th percentile)

*n*, number of patients. %, percent. kg/m^2^, kilograms per square meter

^#^, no statistically significant relation between successful and failed improvement of subjective sporting ability, *p* > 0.05

## Discussion

The main finding of this study was that PFIA leads to a subjective improvement of knee function enabling a high return to sports rate of 94% in the successfully treated patients and 76% of the entire patient cohort including failures. The statistical improvement of all measured clinical and activity scores corresponds with an improved subjective ability to perform low-impact sports, as well as a higher frequency of sport sessions and level of sports postoperatively. Our secondary hypothesis was rejected, as we were not able to detect factors that predict the postoperative ability to perform sportive activities in a statistically significant manner.

While the results of this study underscore the positive effect of the treatment and comprehensively document the consequential improvement of activity and return to sports, the extent of improvement remains limited. Though the results produced in our study are of substantial clinical interest, the minimal clinically relevant difference for the WOMAC score of 15 points determined in a validation study in total knee arthroplasty (TKA) was not surpassed with a preoperative-to-postoperative delta of 10 in our transformed WOMAC results and the smallest detectable difference of 1.16 point in the Tegner activity scores was narrowly missed with a preoperative-to-postoperative delta of 1.13 point in our results [25]. Furthermore, a complete liberation from pain according to the VAS pain scale could not be achieved across the patient cohort by the surgery, observing persistent pain with a median intensity of two points. This is noteworthy, as pain was identified to be the most common barrier to return to sports after arthroplasty of the lower extremity, according to a large retrospective study by Wylde et al. [34]. While Tegner activity scale results range below the average of 5.7 established for a healthy population of comparable age [2], the average patient treated with PFIA is able to participate in low-impact sports such as jogging on even ground twice a week, as well as cycling and cross country skiing, as correlated to a Tegner scale of 4 [3]. This may realistically reflect the sportive demands of the average patellofemoral OA patient, who has been limited in the participation of pivot and contact sports due to the severity of the primary knee injury for years prior to surgery. Nevertheless, these results have to be considered, when preoperatively discussing realistic outcomes of PFIA with more competitive athletes suffering from patellofemoral OA. Furthermore, our patient collective is not involved in high-impact sports (Table 3). However—while this may require preoperative expectation management of young and active patients—it reflects the general clinical guidelines for sports participation after knee arthroplasty [8]. After patellofemoral arthroplasty, only sports with a moderate risk profile for secondary implant damage—characterized by low direct impact and low extensor loads—are recommended [6, 8, 21]. Furthermore, the results need to be put into perspective for the active patient: While 94% of the non-failing patients returned to an equal or higher level of sports after PFIA treatment, only 74% reported an improved subjective sporting ability.

**Table 3:** Sporting activity across the patient cohort

Sport disciplines	Percentage of patients
1. Biking	53%
2. Fitness	24%
3. Hiking	16%
4. Swimming	15%
5. Skiing	13%
6. Gymnastics	13%
7. Jogging	8%
8. Yoga	8%
9. Nordic walking	6%
10. Mountain climbing	5%
11. Pilates	3%
12. Tennis	2%
13. Baseball	2%
14. Golf	2%

Participation in various sport disciplines was evaluated preoperatively

The share of the patient collective practicing each type of sport is shown in %

While the return to activity following UKA [14, 20, 30, 33] and TKA [4, 19, 33, 34] is well described, the literature on patellofemoral arthroplasty is mainly limited to outcome studies investigating implant survival, complications, and clinical scores [10, 11, 15, 27]. Only one clinical study by Shubin-Stein et al. assessed the return to activity following patellofemoral arthroplasty [24]. While this work confirms the results of our study, reporting a high rate of return to activity of 72% following patellofemoral arthroplasty, it describes a lower return to the same or higher level of sports of only 53% [24]. Though comparability may be limited due to differences in the size and composition of the study population (*n* = 39, 84% female) and difference in the implant design used, the study independently confirms patellofemoral arthroplasty to be a viable treatment option for the young active patient with isolated patellofemoral OA [24].

When analyzing return to sports after UKA of the tibiofemoral joint, a systematic review found RTS rates varying between 75 and 98% in studies with a comparable definition of RTS [33]. Analyzing studies after UKA reporting a comparably high RTS rate of 95%, the patient cohort participated mainly in low-impact sports characterized by continuous repetitive load (hiking, walking, swimming, and cycling) [20]—similar to our analysis after PFIA. Data from a metanalysis furthermore indicate that RTS rates after UKA are high in low-impact sports (93%) but limited in high-impact sports (35%) [33]. It was furthermore reported that the ability to compete in high-impact sports is compromised after UKA surgery [20]. While our study could not identify factors predictive for a successful RTS after PFIA, Pietschmann et al. could identify young age to be associated with a superior RTS rate following UKA in a collective of 131 patients [23]. Thus, in summary, the results for RTS following PFIA seem comparable to the RTS data after UKA treatment.

With the trend in surgery shifting to less invasive treatments, the results of modern PFIA treatment, nevertheless, have been benchmarked against TKA, the established treatment for OA in the knee joint [17, 19]. Theoretical advantages for the return to sports and activity after PFIA compared to TKA include a preservation of natural biomechanics of the knee joint due to bone sparing surgery. Indeed, biomechanical data from Vandenneucker et al. [29] show that—in contrast to non-physiologic conditions in the patellofemoral joint after TKA [26]—patellofemoral arthroplasty with concomitant patellar resurfacing can sustain the physiologic kinematics of the patellofemoral joint. While 97% of the patients treated with TKA for patellofemoral arthritis report good or excellent clinical outcomes [19], RTS rates are lower after TKA surgery, ranging from 73 to 86% in comparable study designs with a similar definition of RTS [33, 34]. In line with our findings after PFIA, patients tend to reduce participation in high-impact sports after TKA and adopt lower impact activities. A systematic review reports RTS rates for low-impact sports at 94%, while only 43% of the patients return to high-impact sports [33]. Interestingly, Dahm et al. could show in a retrospective study comparing PFA and TKA in the treatment of patellofemoral OA that PFA patients—while generally showing an improved return to activity—also tend to return to more high-impact sports than matched TKA patients [4]. We did not specifically analyze the time needed for RTS, the number of sports per patient, or subjective satisfaction in their respective sports in PFIA in our study. However, other studies comparing TKA to unicompartmental treatment showed that TKA yields inferior results compared to UKA with only 0.2–1.0 compared to 1.1–4.6 sports per patient, respectively [33], a slower RTS after TKA compared to UKA [9], and an inferior subjective satisfaction after TKA compared to UKA [32]. While similar complication rates are reported for both procedures in isolated patellofemoral OA [5], the unicompartmental PFIA provides unquestionable advantages over TKA including less morbidity, faster rehabilitation, shorter intraoperative tourniquet intervals, and preservation of the bone stock of the tibiofemoral joint, allowing for uncompromised secondary conversion to TKA [4, 28, 31].

### Limitations

While this study does demonstrate interesting findings, it is not without limitations. Surgery was performed with a single PFIA prosthesis, so generalization to treatment with patellofemoral arthroplasty may be limited. Regarding the non-comparative aspect of treating patients in a single reference center for patellofemoral disease, the selection bias may also play a role as the patient cohort may not represent the general population. To precisely evaluate RTS in successful PFIA treatment, 14 patients were excluded for undergoing TKA or revision surgery during the follow-up interval. This potentially introduces a further selection bias, but avoids confounding by TKA results. Furthermore, no radiological follow-up was conducted to evaluate the progression of tibiofemoral arthritis potentially impairing return to sports and activity following PFIA. Due to the lack of a validated score evaluating return to sports in patellofemoral OA in the literature, a query was composed by the authors. While it was attempted to reflect all important aspects in this query, the comparability to reports in the literature may remain limited. Ultimately, further long-term follow-up is needed to determine whether increased sporting activity negatively affects PFIA survival in terms of early wear and loosening of the prosthesis. This is especially critical for PFIA, as patients with isolated patellofemoral OA tend to be younger and more active than patients with tricompartmental OA [4, 11, 13, 16].

## Conclusion

PFIA is a valid treatment option for the active patient with end-stage isolated patellofemoral OA. Reliable improvements in knee function, pain, and participation in low-impact sports were found. However, patient expectations should be appropriately managed preoperatively, as the extent of improvement after surgery may be limited.

## Abbreviations

BMI: Body mass index
DFO: Distal femoral osteotomy
MPFL: Medial patellofemoral ligament
OA: Osteoarthritis
PFIA: Patellofemoral inlay arthroplasty
RTS: Return to sports
TKA: Total knee arthroplasty
UKA: Unicompartmental knee arthroplasty
UKA: Unicompartmental knee arthroplasty
VAS: Visual analog scale
WOMAC: Western Ontario and McMaster Universities Osteoarthritis Index
